# BrainCycles: Experimental Setup for the Combined Measurement of Cortical and Subcortical Activity in Parkinson's Disease Patients during Cycling

**DOI:** 10.3389/fnhum.2016.00685

**Published:** 2017-01-10

**Authors:** Maciej Gratkowski, Lena Storzer, Markus Butz, Alfons Schnitzler, Dietmar Saupe, Sarang S. Dalal

**Affiliations:** ^1^Department of Computer and Information Science, University of KonstanzKonstanz, Germany; ^2^Institute of Clinical Neuroscience and Medical Psychology, Medical Faculty, Heinrich Heine University DüsseldorfDüsseldorf, Germany; ^3^Center of Functionally Integrative Neuroscience, Department of Clinical Medicine, Aarhus UniversityAarhus, Denmark; ^4^Zukunftskolleg and Department of Psychology, University of KonstanzKonstanz, Germany

**Keywords:** DBS, LFPs, EEG, cycling, ergometer, freezing of gait, Parkinson's disease

## Abstract

Recently, it has been demonstrated that bicycling ability remains surprisingly preserved in Parkinson's disease (PD) patients who suffer from freezing of gait. Cycling has been also proposed as a therapeutic means of treating PD symptoms, with some preliminary success. The neural mechanisms behind these phenomena are however not yet understood. One of the reasons is that the investigations of neuronal activity during pedaling have been up to now limited to PET and fMRI studies, which restrict the temporal resolution of analysis, and to scalp EEG focused on cortical activation. However, deeper brain structures like the basal ganglia are also associated with control of voluntary motor movements like cycling and are affected by PD. Deep brain stimulation (DBS) electrodes implanted for therapy in PD patients provide rare and unique access to directly record basal ganglia activity with a very high temporal resolution. In this paper we present an experimental setup allowing combined investigation of basal ganglia local field potentials (LFPs) and scalp EEG underlying bicycling in PD patients. The main part of the setup is a bike simulator consisting of a classic Dutch-style bicycle frame mounted on a commercially available ergometer. The pedal resistance is controllable in real-time by custom software and the pedal position is continuously tracked by custom Arduino-based electronics using optical and magnetic sensors. A portable bioamplifier records the pedal position signal, the angle of the knee, and the foot pressure together with EEG, EMG, and basal ganglia LFPs. A handlebar-mounted display provides additional information for patients riding the bike simulator, including the current and target pedaling rate. In order to demonstrate the utility of the setup, example data from pilot recordings are shown. The presented experimental setup provides means to directly record basal ganglia activity not only during cycling but also during other movement tasks in patients who have undergone DBS treatment. Thus, it can facilitate studies comparing bicycling and walking, to elucidate why PD patients often retain the ability to bicycle despite severe freezing of gait. Moreover it can help clarifying the mechanism through which cycling may have therapeutic benefits.

## 1. Introduction

Recently, it has been demonstrated that bicycling ability remains surprisingly preserved in Parkinson's disease (PD) patients. In the first report on this phenomenon (Snijders and Bloem, 2010), a video of a 58-year-old man with a 10-year history of idiopathic PD suffering from severe freezing of gait (FOG) is presented. The patient had difficulties initiating gait, which resulted in forward festination and eventually in a fall to the ground. He was able to perform only a few steps when provided with a visual cue and the axial turning was not possible at all. However, the patient had no apparent problems riding and controlling a bike. Immediately after hopping off the bike the FOG episode recurred. Follow-up reports by these investigators showed that most PD patients, with and without FOG, maintain the ability to bicycle despite severe walking deficits (Snijders et al., 2011, 2012). It has been also demonstrated that the loss of bicycling ability early in the progression of disease strongly supports a diagnosis of atypical parkinsonism rather than PD (Aerts et al., 2011).

It has long been established that PD patients have abnormal basal ganglia function. As the basal ganglia are thought to be critical for all types of locomotion, the observation that bicycling and walking are differentially impacted in FOG is surprising. Snijders et al. (2011) propose possible explanations for this phenomenon: The bicycle's rotating pedals may act as an external pacing cue for the legs. Alternatively, gait and other activities like cycling, which involve moving the legs, might be differentially affected in PD. One may also speculate that the special conditions of bicycling, e.g., continuous resistance and angular momentum of the pedals, may provide feedback that is substantially different from walking. Bicycling has also been recently promoted as a viable therapy for PD (Mohammadi-Abdar et al., 2016), with evidence emerging that it may stimulate improvements in motor control (Ridgel et al., 2009, 2015) and cognitive performance (Alberts et al., 2011; Ridgel et al., 2011), as well as reduce severity of tremor, bradykinesia (Ridgel et al., 2012) and of orthostatic hypotension (Ridgel et al., 2016).

The neural mechanisms behind these phenomena are however not yet understood. One of the reasons is that investigation of neuronal activity during pedaling has been limited up to now to functional imaging and scalp EEG studies. Functional imaging restricts the temporal resolution of analysis, and scalp EEG is focused on cortical activation. Christensen et al. (2000) examined bicycling movements with PET in healthy subjects, observing activation of many typical motor structures (primary motor cortex, supplementary motor area, cerebellum), but notably not basal ganglia. Fukuyama et al. (1997) used SPECT to determine involvement of the basal ganglia in walking in healthy subjects, along with primary motor cortex, supplementary motor area, and cerebellum. SPECT, however, reflects the summation of all brain activity over several minutes and can therefore not reveal oscillatory activity or connectivity patterns. A recent study in dystonia patients measured local field potentials (LFPs) from the basal ganglia while they walked on a treadmill, and found increases in theta (4–8 Hz), alpha (8–12 Hz), and gamma (60–90 Hz) power compared to rest, while beta (15–25 Hz) power was markedly reduced (Singh et al., 2011). Importantly, no power differences were noted between sitting and standing positions, reducing the likelihood that the seated configuration of bicycling could play a significant role in the context of the bicycling ability phenomena in PD patients. The neural activity during walking has been however mostly investigated using scalp EEG in healthy subjects. Sipp et al. (2013) found increased power in the theta band in anterior cingulate, anterior parietal, superior dorsolateral-prefrontal, and medial sensorimotor cortex as well as decreased beta power in sensorimotor cortex during walking on a balance beam compared with treadmill walking. Wagner et al. (2014) found that mu, beta, and lower gamma rhythms in premotor and parietal cortices are suppressed during conditions that require an adaptation of steps in response to visual input. In another study (Wagner et al., 2016) they found two distinct beta band networks active during gait adaptation to shifts in the tempo of an auditory pacing cue. Mean beta band power was suppressed in central midline and parietal regions and increased in medial prefrontal and dorsolateral prefrontal cortex. The beta suppression may be related to initiation and execution of movement, while the prefrontal beta increase to cognitive top-down control. Seeber et al. (2014) found that during active walking the upper mu (10–12 Hz) and beta (18–30 Hz) oscillations were suppressed compared to upright standing and the significant beta ERD activity was located focally in central sensorimotor areas. They also found that low gamma (24–40 Hz) amplitudes were modulated related to the gait phase. The gait phase dependent gamma modulation may be linked to sensorimotor processing or integration, while the decrease of beta may reflect the suppression of an inhibitory network that enables voluntary movement. In Seeber et al. (2015) they reported increased high gamma (60–80 Hz) amplitudes during human upright walking as compared to standing. The high gamma activity was located focally in central sensorimotor areas and was increased during the gait cycle, which may facilitate motor processing.

The first scalp EEG study of cortical activity as a function of instantaneous pedaling (Jain et al., 2013) reported that beta power over the motor cortex was significantly reduced in active pedaling as opposed to passive pedaling. Furthermore, they demonstrated a relationship between EEG power and EMG power of various leg muscles as a function of pedal position. Similar results were presented by Wagner et al. (2012) in a study of cortical activity related to lower limb movements in robot assisted gait. Power in the mu and beta bands over central midline areas was significantly reduced during active compared to passive walking and the decrease was dependent on gait cycle phases.

Recently, it has been shown using scalp EEG that bicycling relative to walking has a stronger sustained cortical activation and less demanding cortical motor control within the movement cycle (Storzer et al., 2016). This is probably due to the fact that walking demands more phase-dependent sensory processing and motor planning, because each leg is independent in altering stance and swing movement phases. In bicycling, pedals are locked to each other, imposing continuous movement of both legs.

No study to date has recorded deep brain activity during bicycle pedaling. Thus, there is a need for further investigation of cortical and deep brain structures to understand both the mechanism through which cycling ability is preserved in PD patients and the mechanism through which cycling may have therapeutic benefits for them. Electrodes implanted for deep brain stimulation (DBS) therapy in PD patients provide the unique chance to directly record basal ganglia activity.

DBS is an established therapeutic strategy that, for PD patients, involves neurosurgical implantation of electrodes directly in the basal ganglia to allow stimulation with electric current, somewhat analogous to a cardiac pacemaker. If externalized, the same electrodes can be used to also record LFPs, providing an opportunity to measure basal ganglia activity during motor and cognitive behavior.

In this paper, we present an experimental setup that we call *BrainCycles*. It allows combined investigation of basal ganglia LFPs and scalp EEG in conjunction with physiological and performance parameters during bicycling tasks in patients with implanted DBS electrodes. Furthermore, example data from pilot recordings with PD patients are presented.

## 2. Materials and methods

### 2.1. Experimental setup overview

**Figure 1:**
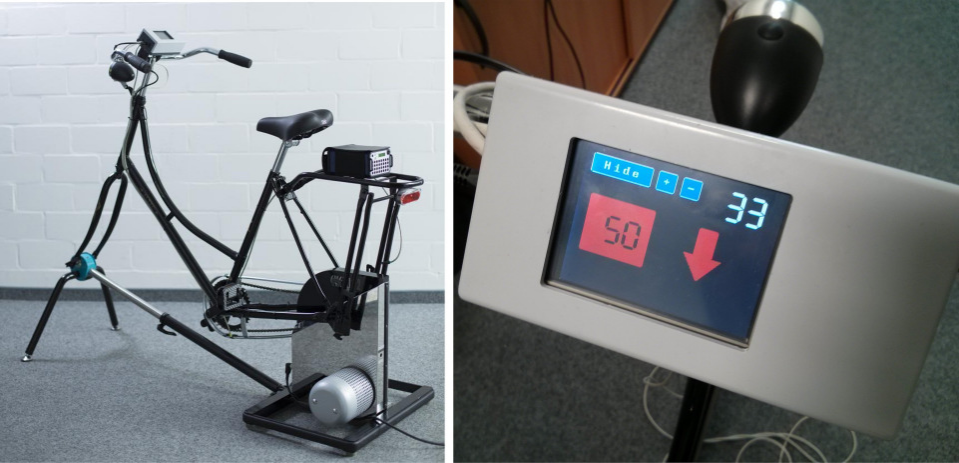
**The BrainCycles experimental setup**. **(Left)** Classic Dutch frame and EEG amplifier mounted on the Cyclus2 ergometer. **(Right)** Handlebar display presenting information about the current and the desired target cadence in rpm.

The BrainCycles setup is a modified version of the Powerbike simulator, which was developed for data acquisition, analysis, and visualization of performance parameters in endurance cycling (Dahmen et al., 2011). The full Powerbike software suite incorporates cyclist and bicycle management, synchronized videos of cycling routes, an electronic gear shifter, and the recording and visualization of various performance parameters during a ride like speed, cadence, power, heart rate, and height profile. For the present experimental setup, the Powerbike's capabilities to record bicycling performance parameters as well as to actively control pedal resistance are used. Additionally, the setup was extended with a device for real-time measurement of pedal position. Custom software was developed for the control of the acquisition of the EEG, EMG and other physiological parameters important for experiment protocols involving PD patients. The main hardware component of the setup is a Cyclus2 ergometer (RBM elektronik-automation GmbH, Germany) with an eddy current brake. The brake force can be controlled through a serial port at a rate of 20 Hz using a custom-made software interface. A classic Dutch-style bike frame is mounted on the ergometer brake. The frame, saddle and handlebar were chosen taking the special needs of PD patients into consideration. They ensure that the patients are able to easily get on and get off the bike simulator and have a comfortable sitting position (see Figure 1).

An Arduino Due board (Arduino LLC, USA) controls the sensors that assess the crank phase angle and a TFT-display mounted on the handlebar. The Due board includes an Atmel SAM3X8E ARM Cortex-M3 microcontroller and features 54 digital input/output pins, 12 analog inputs, 4 hardware serial ports, an 84 MHz clock and 2 digital-to-analog converters (DACs). A vast number of extension boards (shields) and libraries for interfacing to a wide range of hardware is available for the Arduino platform. Thus, the board is especially suitable for easy and fast prototyping.

In this setup a TFT/SD shield (SainSmart, USA) is used to interface with a 3.2 TFT LCD touch-display (SainSmart, USA). The display is mounted on the handlebar and is used to present information and feedback for participants during experiments. For example, the display can show current and desired target cadence in revolutions per minute (rpm). If the target cadence is not being met, a colored arrow is shown, signaling the patient to pedal faster or slower. The color (red or yellow) of the arrow indicates the magnitude of deviation from the target cadence, with the thresholds adjustable by the experimenter.

The data acquisition in the BrainCycles setup is managed with the help of the OpenBCI software framework (Durka et al., 2012). OpenBCI is an open source software modular framework for brain-computer interfaces. The architecture of the framework is based on a centralized modular approach, where different modules communicate through a central multiplexer. The framework includes a module for online communication with signal acquisition hardware and a graphical module for signal review. The OpenBCI's tags manager module can be used to annotate the electrophysiological data with events like FOG episodes or start/stop signals, which are used to instruct participants to start or stop pedaling. The open source character of the framework enables the integration of cycling performance measures within the data stream. For example, twice per pedal revolution, data such as current brake force and cadence are saved as tags in the data stream. The analysis module enables the realization of feedback loops, e.g., in order to control pedal brake force depending on the parameters of electrophysiological signals like EEG or EMG.

### 2.2. Data acquisition

All electrophysiological data is acquired by a 32-channel TMSi Porti amplifier (TMSi, Enschede, The Netherlands). This lightweight, compact amplifier is battery-powered and can therefore be worn by the patient on a belt and used for recording signals “untethered” for comparison of walking and pedaling conditions. It has a maximum sampling rate of 2048 Hz, and, unlike most other portable devices, is therefore capable of capturing high frequency oscillations (100–500 Hz) particularly relevant for basal ganglia pathology in PD (Özkurt et al., 2011; Hirschmann et al., 2016). Additionally, it has several auxiliary channels, which can be used for the recording of performance data from the ergometer and other physical measurements. The amplifier provides 24 unipolar, 4 bipolar as well as 4 auxiliary channels. The 32-channels can be used to record from basal ganglia implants, which have 4–8 electrode contacts each, typically placed bilaterally for a total of 8–16 intracerebral channels. Scalp EEG can be recorded with an electrode montage optimized for capturing sensorimotor cortex activity (e.g., Fp1, Fz, Cz, C3, C4, and reference), along with bilateral EMG of the tibialis anterior (TA), rectus femoris (RF), and biceps femoris (BF) leg muscles. EMG and scalp EEG are recorded with actively shielded cables, which reduce sensitivity to motion artifacts and are therefore particularly suitable for recording during locomotion tasks. Furthermore, respiration rate, ECG, knee flexion and foot pressure can also be monitored. TMSi electronic goniometers and footswitches (TMSi, Enschede, The Netherlands) were successfully tested with the setup.

### 2.3. Measurement of LFPs

DBS treatment of PD involves the implantation of electrodes for deep brain stimulation in the subthalamic nucleus (STN) or the globus pallidus interna (GPi). At many DBS clinics, the electrodes are implanted in a first step, and the stimulator device a few days later in a second step, allowing the chance to record LFPs from the externalized electrodes. Light physical activity is usually manageable for PD patients shortly after the implantation of electrodes. Thus, it is possible to record LFPs while the PD patient is pedaling a stationary bicycle in a laboratory environment. The LFPs can be recorded unipolarly or bipolarly using selected pairs of DBS electrode contacts. Bipolar measurements or bipolar digital rereferencing should be used in order to reduce contamination from distant sources as well as movement artifacts. Those could degrade true local LFPs, bias power spectral estimates, or influence coherence and correlation estimations between different brain regions (Whitmore and Lin, 2016). We have successfully tested Boston Scientific Vercise and St. Jude Medical Infinity DBS systems with the BrainCycles experimental setup. Both of them offer 8 contacts per electrode. Using directional leads they allow for stimulation and measurement in directions orthogonal to the lead trajectory.

### 2.4. Measurement of pedal position

One of the requirements of the BrainCycles setup is to provide a means to investigate possible relationships between EEG power and EMG power of various leg muscles as a function of pedal position. A custom made electronic circuit encodes the current crank position as an analog signal proportional to the angular position of the pedals. This analog signal is acquired together with electrophysiological signals using the auxiliary input of the EEG amplifier. An important advantage of this approach is that this eliminates the need to coregister data prior to analysis. Furthermore, the knowledge of the real-time pedal position opens new possibilities for experimental protocols with tasks or control parameters depending on the instantaneous position of the pedals. For example, the pedaling resistance at the push-down phase of the pedal motion could be independently set for each leg.

The main part of the rotary encoder are two forked light barriers consisting of infrared LEDs and phototransistors. The barriers are mounted next to the front chainring so that the teeth of the chainring pass through the light barriers, and the distance between the light axes of the barriers is smaller than the width of the teeth. The first light barrier acts as a key that changes the state when a tooth first breaks and then releases the light barrier. Thus, an inversed impulse is created every time a tooth passes through the barrier and temporarily interrupts the light. The falling edge of the impulse invokes a hardware interrupt in the microcontroller. The microcontroller counts each such event. The increase of the angular position of the cranks can be derived from this count and the number of teeth on the chainring. The orientation of the crank rotation can be determined with the help of the second barrier. If the pedaling is forward, the second barrier will be open at the time the tooth is entering the first barrier. If the pedaling is backwards, the second barrier will be closed.

The angular resolution of such encoder is directly proportional to the number of teeth on the chainring, because the teeth on the sprocket are equidistantly distributed. In our particular setup, the chainring has 42 teeth and thus the angular resolution is 360°/42 = 8.57°. In order to know the absolute position of the pedals a reset signal at position 0° is needed. The reset signal is provided by mounting a magnet on the crank and a magnetically actuated reed switch at position 0°, which in our case is the top position of the right pedal. The absolute position of the pedals is then converted to an analog signal by the DAC integrated on the Arduino Due board and connected to the auxiliary input of the EEG amplifier. The DAC output and the auxiliary input of the Porti amplifier are galvanically separated with an optocoupler for an additional layer of patient safety and to prevent ground loop interference.

### 2.5. Example data

In order to demonstrate the utility of the setup, sample data from pilot recordings is presented. The shown data were recorded from a 56-year-old patient, who was diagnosed with PD in 2011 with motor symptoms predominantly on the right side. The patient had opted for bilateral DBS therapy (Boston Scientific Vercise system with 8 contacts per STN), and was recorded 1 day after the implantation of DBS electrodes while off any dopaminergic medication. Custom-made connectors were used to connect the DBS electrodes with the EEG amplifier.

The recording protocol consisted of 30 intermittent rest and pedaling periods guided by acoustic signals. The patient was provided with start and stop signals in form of a tone of 500 ms duration and frequency 1000 Hz in case of the start signal and 1500 Hz for the end signal. The duration of the rest and pedaling phases were around 10 seconds. The patient was instructed to pedal at a relaxed pace of his own preferred cadence. The brake force of the ergometer was constantly kept at a low level of 30 N. Individual gel-based electrodes were placed at Pz, Oz, and P3 to capture signals from somatomotor areas. The bandage left after the implantation of electrodes did not allow placement of electrodes more anterior. Subsequently, the recorded signals were digitally rereferenced to a bipolar montage of Pz-P3 and Pz-Oz. A water-based ground electrode (TMSi, Netherlands) integrated in a wristband was used. Bipolar surface EMG activity of TA, BF, and RF was recorded bilaterally using disposable Ag/AgCl electrodes. Electrodes were placed 2 cm apart on the belly of each muscle. EMG, scalp EEG and LFPs were recorded with actively shielded cables compensating for movement artifacts.

In previous studies, modulation of cortical oscillatory activity in the beta band during transitions between rest and pedaling conditions was observed (Storzer et al., 2016). Similarly, Wagner et al. (2012) and Seeber et al. (2014) showed a beta band decrease during walking compared to rest. Thus, we hypothesized that this modulation should also be seen in deeper brain regions. The recorded data were processed in Matlab (Mathworks, Inc., Natick, MA) using the FieldTrip open source Toolbox (Oostenveld et al., 2011). The bipolar LFP channel with the highest resting beta activity was chosen for further analysis. LFPs and EEG data were bandpass filtered with cutoff frequencies of 13 and 33 Hz. Next, the filtered data were Hilbert-transformed in order to compute their envelopes. The envelope samples from cycling and rest periods were statistically compared using Mann-Whitney *U*-test for equal medians (ranksum function, Matlab). Furthermore, the envelope signals were averaged taking 5 s of data before and 10 s of data after each movement initiation. Both ongoing and averaged envelope signals were filtered by a moving average filter of length 2500 samples, which represents around 1.2 s of data.

## 3. Results

**Figure 2:**
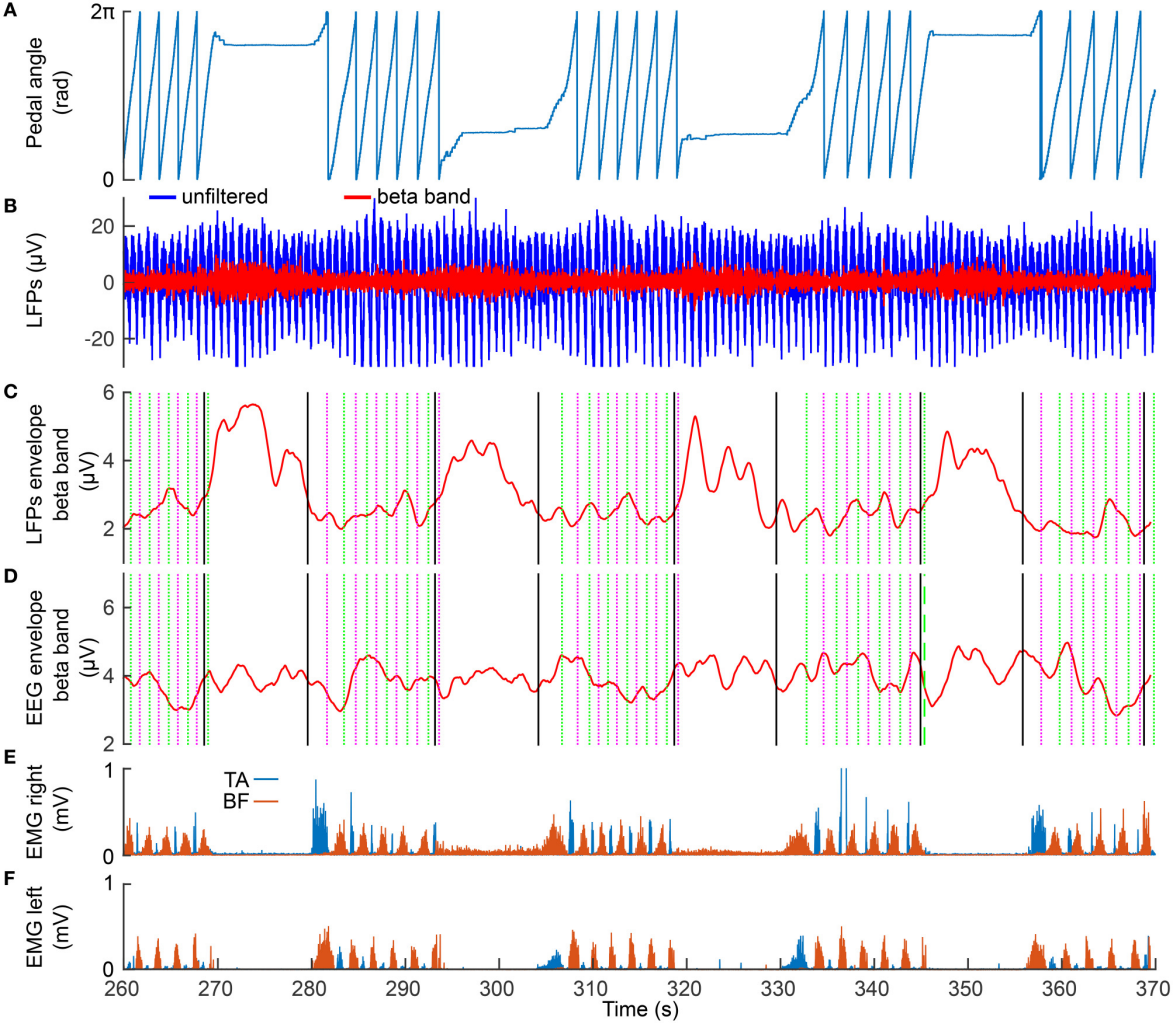
**Modulation of the beta band in the LFPs (left STN) and EEG data (Pz-Oz)**. **(A)** the recorded pedal angle. **(B)** the unfiltered (blue curve) and filtered (red curve) data recorded from the left STN. **(C,D)** the envelopes of filtered LFPs and EEG data. The solid black lines represent start and stop signals. The magenta and green dotted lines represent the events of the right pedal crossing the top and bottom positions, respectively. **(E,F)** EMG signals recorded from TA and BF in the right and left legs.

**Figure 3:**
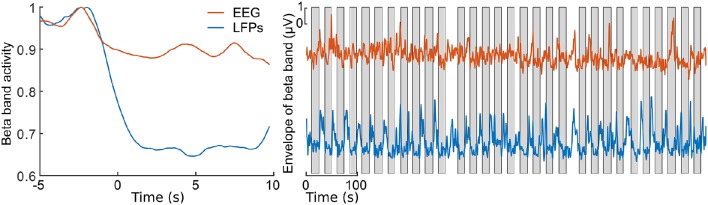
**Envelopes of the beta band of the EEG Pz-Oz signal (red) and the LFPs of the left STN (blue)**. At **left**, the averaged and normalized envelopes are presented. The averaging was locked to the movement initiation at *t* = 0 s and the curves are normalized to their maximum value. At **right**, ongoing envelopes are presented. The gray boxes represent periods of pedaling. All the envelopes were filtered with a moving average filter of length 2500 samples (1.2 s).

Figure 2 presents 110 seconds of continuously recorded data including the pedal angle (Figure 2A), the LFPs from the left STN (Figures 2B,C), the envelope of the beta band of the EEG Pz-Oz signal (Figure 2D) and the muscle activity of TA and BF (Figures 2E,F). Beta band activity in the STN decreased upon initiation of pedaling and rebounded after termination. This can already be seen in the filtered LPF data and its envelope (see Figures 2B,C). The beta increase is visible upon return to resting, when muscle activity is minimal. Thus, it is unlikely that the increase in the beta band is due to movement artifacts. The beta modulation can also be observed from the scalp EEG data (Pz-Oz) when averaged relative to movement initiation (see Figure 3). The Mann-Whitney *U*-test showed that the difference in the beta band between cycling and resting condition, both in EEG and LFPs is statistically significant (*p* < 0.001).

## 4. Discussion

In this paper we presented an experimental setup for combined recording of cortical and deep brain oscillations during pedaling. We showed that direct recordings from the STN during bicycling are possible in a laboratory environment and that combining these signals with data from an ergometer is feasible, safe, and not prone to excessive noise or artifacts. Visual inspection suggests that the recorded signals have a very high SNR. The reduction of the beta power in the STN during bicycling is visible even in the raw data. A similar effect could be observed in the surface EEG. However, for the latter the change of the amplitude is much smaller and only visible after averaging the EEG locked to the movement initiation. We did not observe strong movement related artifacts in our LFPs and EEG data. However, data recorded at higher cadences and/or at higher resistive forces could exhibit strong rhytmic, movement related artifacts. Such artifacts could be minimized using a template regression procedure applied previously to EEG data recorded during walking and running (Gwin et al., 2010).

The presented experimental setup provides means to directly record basal ganglia activity not only during cycling but also during other movement tasks in patients who have elected DBS treatment. Thus, it can facilitate studies comparing bicycling and walking, to elucidate why PD patients often retain the ability to bicycle despite severe FOG. Moreover, it can help to clarify the mechanism through which cycling may have therapeutic benefits.

At present, the experiments can be performed between 1 and 6 days after electrode implantation, while the electrode cable is externalized and therefore allows recording of LFPs from the implanted structures. The next generation of DBS systems will potentially allow LFPs recording by the same unit that performs stimulation and storing the data internally for subsequent retrieval via wireless data transfer (Neumann et al., 2016). This will allow recording of data after electrode cables have been internalized, expanding opportunities to record LFPs, and eventually allowing recordings after patients have recovered from the surgical procedure and have become accustomed to the implant. Thus, task-related modulations of oscillatory activity and coupling could be monitored during a cycling training regimen and correlated to therapeutic outcomes. Importantly, several months after implantation, the patients would be able to participate in more strenuous activity, such as the forced pedaling regimens described in Alberts et al. (2011) and Ridgel et al. (2012).

**Figure 4:**
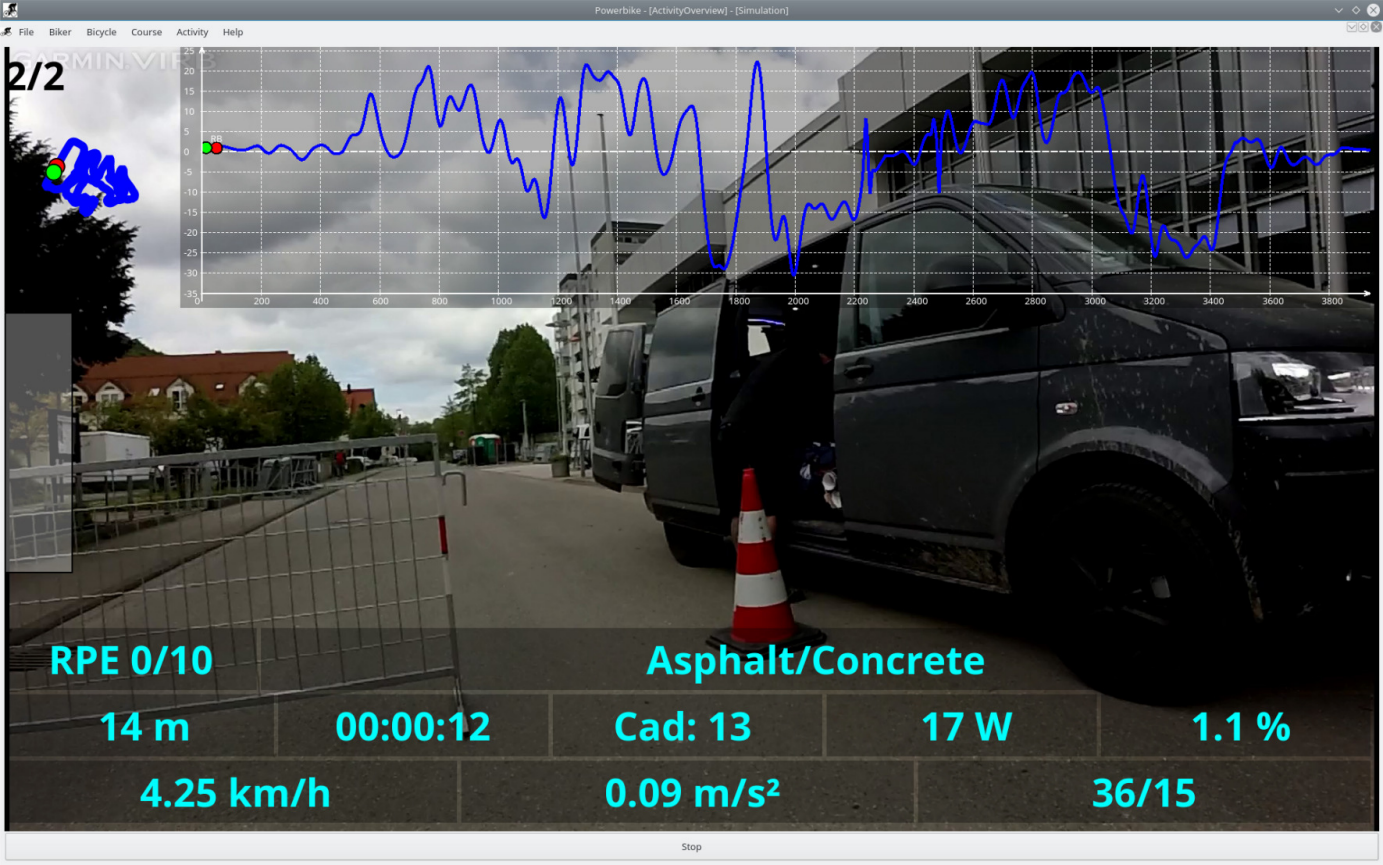
**Screenshot from a Powerbike simulation of a ride on a real-world track**. The playback speed of a video recorded on the track is controlled by the pedaling speed and the ergometer brake force simulates the incline of the track. The software enables the acquisition of various cycling performance parameters like cadence, pedaling power, cycling speed, acceleration, and distribution of the power over the pedaling cycle. The blue curves in the top represent the map and the slope profile of the simulated track. The Powerbike simulation software could serve as a virtual reality environment for the BrainCycles setup, enabling studies with visual feedback or simulating real-world bicycle navigation in patients with movement disorders.

The BrainCycle setup is linkable to a virtual reality environment provided by the Powerbike software, that enables simulation of bicycling on a given route, with the speed of pedaling controlling the speed of navigation and real-time modulation of brake force to simulate incline (see Figure 4). This feature can enable several new lines of research involving visual feedback or simulating real-world bicycle navigation in patients with movement disorders. For instance, the BrainCycles setup could be used to investigate visuomotor processing in PD patients. It has been hypothesized that PD is associated with a visuomotor disturbance and PD patients produce exaggerated responses to visual information (Cowie et al., 2010). For example, many patients slow down dramatically or even freeze when attempting to approach narrow doorways. Similarly, FOG episodes can be elicited by unexpectedly appearing obstacles (Delval et al., 2010). The Powerbike's ability to play videos synchronized to pedaling could be used to investigate how such sudden visual cues and obstacles are processed during cycling. Visuomotor processing during walking on a treadmill and cycling on an ergometer could be also compared in a lab environment.

## Ethics statement

The study protocol was approved by the local ethics committee of the Medical Faculty of the Heinrich Heine University Düsseldorf (study number: 4294). All participants gave their prior written informed consent.

## Author contributions

All authors contributed to the design of the experiment setup and revised the manuscript. MG constructed and programmed the BrainCycles setup. LS acquired the data. MG, SSD, and LS were involved in data analysis and interpretation. All authors approved the final version of the manuscript, agreed to be accountable for all aspects of the work and qualify for authorship.

## Funding

This work was generously supported by a grant from the Jacques and Gloria Gossweiler Foundation. Further support was received from the Zukunftskolleg of the University of Konstanz [SSD], Heinrich Heine University Strategic Research Funds [MB], the Open Access Publication Fund of the University of Konstanz, and the research commission of the medical faculty of the Heinrich Heine University [9772562 to MB]. LS was supported by a travel grant from the Boehringer Ingelheim Foundation (BIF).

### Conflict of interest statement

The authors declare that the research was conducted in the absence of any commercial or financial relationships that could be construed as a potential conflict of interest.
